# Enhancing the Reliability of Head Nodes in Underwater Sensor Networks

**DOI:** 10.3390/s120201194

**Published:** 2012-01-31

**Authors:** Hong Min, Yookun Cho, Junyoung Heo

**Affiliations:** 1 School of Computer Science and Engineering, Seoul National University, Seoul 151-742, Korea; E-Mails: hmin@os.snu.ac.kr (H.M.); ykcho@os.snu.ac.kr (Y.C.); 2 Department of Computer Engineering, Hansung University, Seoul 151-742, Korea

**Keywords:** underwater sensor networks, clustering, checkpointing

## Abstract

Underwater environments are quite different from terrestrial environments in terms of the communication media and operating conditions associated with those environments. In underwater sensor networks, the probability of node failure is high because sensor nodes are deployed in harsher environments than ground-based networks. The sensor nodes are surrounded by salt water and moved around by waves and currents. Many studies have focused on underwater communication environments in an effort to improve the data transmission throughput. In this paper, we present a checkpointing scheme for the head nodes to quickly recover from a head node failure. Experimental results show that the proposed scheme enhances the reliability of the networks and makes them more efficient in terms of energy consumption and the recovery latency compared to the previous scheme without checkpointing.

## 1. Introduction

As the amount of underground resources such as known stores of crude petroleum increases, the exhaustion of these resources is also being accelerated. Moreover, some countries that are rich in natural resources put pressure on other countries by limiting the export volume of their resources. Developing alternative types of resources is one solution, but the results so far have not been practical or cost-effective. The ocean, which occupies 70% of the surface of the Earth, has received attention related to resource development, not only for its size but also for its potential.

Underwater Wireless Sensor Networks (UWSNs) are significantly different from Terrestrial Wireless Sensor Networks (TWSNs) in many aspects, such as their high latency, high error probability of each node, and their high communication cost [1]. UWSNs also have some disadvantages including the unavailability of real-time monitoring, limited interaction between an onshore control center and the monitoring instruments, and lack of a mechanism to detect failures quickly [2]. These characteristics make existing TWSNs unsuitable as UWSNs. Specifically, the major concerns in UWSNs are reduced energy use and reliable communication, since the sensor nodes are powered by batteries, which are difficult to replace or recharge in underwater environments and have a high error rate. Therefore, node clustering, which has been widely studied in UWSNs, is an effective technique to improve energy efficiency and to simplify the network architecture [3]. In addition, numerous studies have attempted to improve the reliability of the communication based on clustering protocols.

A cluster-based UWSN is similar to a TWSN. The clustering protocols elect a head node, and other nodes transmit sensing data to their head nodes. The head node transmits the collected data to an underwater sink that is specially designed to communicate with the surface sink. In its role, the head node is more important in UWSNs than other nodes. If a cluster head failure is not accurately detected, it will unnecessarily execute a fault recovery process (head node reelection or network re-clustering) and thus, waste energy in the sensor network. To avoid this, it is important to be able to accurately detect head node failures [4].

To reduce latency and improve reliability during the recovery process, we propose a checkpointing scheme, which stores the state of the head node and repairs the head node when it fails. The head node sends routing information and collected data to the backup nodes that periodically save this information. If a head node experiences a transient fault, one of the backup nodes detects the head node failure and becomes the new head node. The head node can quickly recover from a transient fault by omitting the reelection of a head node and by preventing the loss of the collected information. Even if a head node experiences a permanent fault, a backup node becomes the new head node immediately.

The balance of this paper is organized as follows. Section 2 describes the architecture and background knowledge pertaining to underwater sensor networks. In Section 3, we describe some related works to improve the reliability of UWSNs. In Section 4, we propose the recovery scheme for a head node failure. In Section 5, we evaluate the proposed scheme. Finally, we provide some concluding remarks in Section 6.

## 2. Underwater Sensor Networks

### 2.1. Underwater Channels

TWSNs use radio frequency waves, but UWSNs rely on physical means like acoustic waves to transmit signals. In terms of protocols, the networks stacks of TWSNs are not suitable for UWSNs since low bandwidths and large latency result in long end-to-end delays [5]. Acoustic signals have unique characteristics. First, acoustic signals tend to have high transmission losses due to attenuation and geometric spreading. The attenuation is induced by absorption due to the conversion of the acoustic energy into heat. Geometric spreading means the propagation of sound energy because of the expansion of the wave front. Second, it is easy for acoustic signals to be affected by noise. Noise includes man-made noise caused by shipping activities and ambient noise caused by natural phenomena such as tides, currents, and storms.

The speed of sound in sea-water is a function of the temperature, salinity and depth, as expressed by Equation (1):

(1) $$\begin{array}{ll} C(D,S,T)= & 1448.96+4.59T-5.3\times 10^{-2}T^{2}+2.374\times 10^{-4}T^{3}+1.34(S-35)\\ & +1.63\times 10^{-2}D+1.675\times 10^{-7}D^{2}-1.025\times 10^{-2}T(S-35)-7.139\\ & \times 10^{-13}{\text{TD}}^{3} \end{array}$$

**Figure 1.**
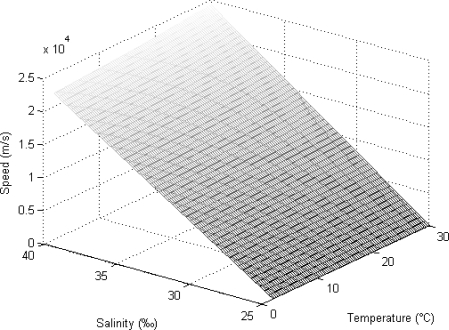
The speed of the acoustic signal.

Here, T, S, and D refer to the temperature in degrees Celsius, the salinity in parts per thousand, and the depth in meters respectively. This equation is valid within the temperature range from 2 to 30 °C, 25 to 40 parts per thousand (‰) salinity and depths from 0 to 8,000 m. Figure 1 shows the change in the speed of the acoustic signal with the temperature and the salinity if the depth is fixed at 50 m. When the temperature of seawater increases, the speed of the acoustic signal also increases steadily. The speed shows a dramatic increase if the salinity increases. The effect of the salinity is higher than that of the temperature.

Equation (2) determines the attenuation of the acoustic signal [A(*d*)] according to the distance (*d*) and the communication frequency (*f*) in the ocean [6]: Here, *d* is the distance (m), *f* is the frequency (kHz) and α(*f*) is the absorption coefficient (dB/km)

(2) $$\begin{array}{c} A(d)={da}^{d},a=10^{\alpha (f)/10}\\ \alpha (f)=0.11\frac{f^{2}}{1+f^{2}}+44\frac{f^{2}}{4100+f^{2}}+2.75\cdot 10^{-4}f^{2}+0.003 \end{array}$$

**Figure 2.**
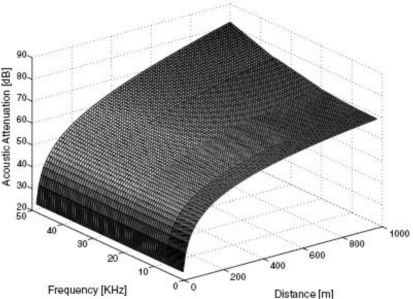
The attenuation of the acoustic signal.

Here, *d* is the distance (m), *f* is the frequency (kHz) and α(*f*) is the absorption coefficient (dB/km) Figure 2 shows a graph of this attenuation with the distance and the communication frequency. In UWSNs, the available bandwidth (under 50 kHz) is severely limited due to the extreme characteristics of the underwater channel. As the distance increases, the attenuation also increases rapidly.

**Figure 3.**
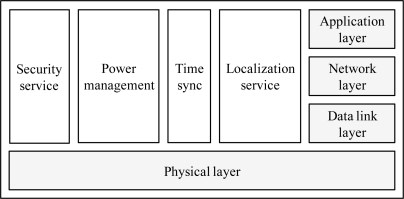
Cross layer protocol stack.

To improve the efficiency of data transmission using the acoustic signal, the design of the network protocol stack is important. A protocol stack for UWSNs should manage power and promote cooperation among the underwater sensor nodes. The protocol stack consists of physical layer, data link layer, network layer, transport layer, and application layer functionalities, as shown in Figure 3.

The security service protects the underwater sensor nodes and their communications. The power management plane is responsible for the network functionalities aimed at minimizing the energy consumption. The time synchronization service adjusts the local time of each sensor nodes to the global time. The localization service is responsible for providing absolute or relative localization information to the underwater sensor node, when needed by the protocol stack or by the application.

### 2.2. Underwater Platforms

**Figure 4.**
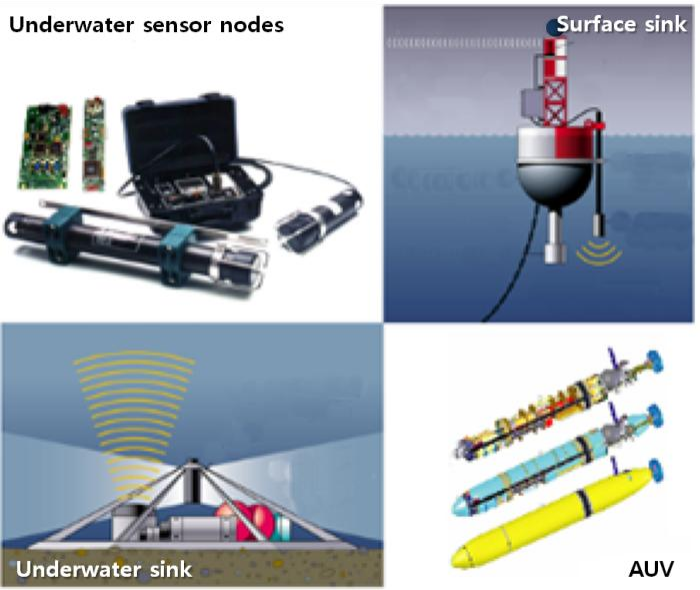
Underwater platforms.

The hardware includes underwater sensor nodes, surface sinks and mobile Autonomous Underwater Vehicles (AUVs), as shown in Figure 4 [7]. Each sensor node is equipped with a processor, memory, storage and an acoustic modem. Nodes also include temperature sensor, pressure sensor, a gyroscope and a camera to monitor their environments [8]. The sensor nodes communicate with each other using a Time Division Multiple Access (TDMA) protocol to avoid signal collisions. The signal propagation speed of an underwater acoustic channel is about 1.5 × 10^3^ m/s, which is much lower than the Radio Frequency (RF) propagation speed (3.0 × 10^8^ m/s) [9]. The data rate is less than 8,000 bits/s because the available bandwidth of underwater acoustic channels is limited and depends on both the transmission range and the frequency [10]. These limitations are caused by the absorption of acoustic signals that operate below 30 kHz. The features of underwater acoustic channels are a long propagation delay, limited available bandwidth and high error probability [11].

An autonomous underwater vehicle (AUV) is a vehicle which travels underwater without requiring commands from an operator. AUVs include sensors to prevent collisions, propulsion devices to move them forward, and batteries to supply power. They collect data from head nodes as they travel among head nodes. A surface sink node is attached onto a floating buoy with satellite or RF capabilities to transmit data to an onshore control center. It aggregates data sent from head nodes by using the acoustic signal. To reduce propagation delay, the surface sink connects to the acoustic modem by wire near the bottom of the ocean. The onshore control center manages and monitors the entire network.

### 2.2. Architecture

UWSNs have a number of limitations, such as their harsh environment, high mobility, long propagation delays and limited communication bandwidth. Under such conditions, communication failures arise more frequently in UWSNs than they do in TWSNs. Therefore, a technique is needed for alternating the direct communication between the sensor nodes and surface station. A clustering or hierarchical protocol can improve scalability, energy efficiency and the lifetime of the network in UWSNs.

**Figure 5.**
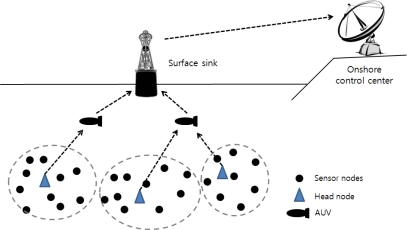
UWSN architecture.

The architecture of underwater wireless sensor networks is presented in Figure 5 [1]. A group of sensor nodes is anchored to the bottom of the ocean. Sensor nodes are interconnected to one or more head nodes via wireless acoustic communication. Head nodes are devices in charge of relaying data from sensor nodes located at the bottom of the ocean to AUVs indirectly or a surface sink directly. AUVs dive between the surface and go to the bottom of the ocean. They communicate and relay the collected data sent from head nodes to the surface sink to reduce the energy consumption of the head nodes and the data transmission latency. The surface sink is equipped with an acoustic transceiver capable of handling multiple communication instances with AUVs. The surface sink also supports a long-rage RF and satellite transmitter to communicate with the onshore control center. The onshore control center collects data from the surface sink and manages the underwater network.

## 3. Related Works

### 3.1. Data Transmission

UWSNs using acoustic signals have limited bandwidth and long propagation delays. There have been many studies to improve the reliability of data transmission under these conditions. The Reliable Routing and Application-based Scheduling protocol (RRAS) [12], which is a priority scheduling approach for multihop topologies, tries to balance between reliability and efficiency at the Media Access Control (MAC) layer. To achieve this goal, it utilizes the NACK-retransmission mechanism that delays the request of lost packets for an additional retransmission period. It divides time lots by the data transmission period and the retransmission period to reduce control frame handshaking and improve the throughput.

In [13], Multiple-path Forward Error Correction (M-FEC) reinforces the existing Multi-Path Communication (MPC) in terms of reliability and energy efficiency. In previous MPC scenarios, receivers requested the retransmission of the lost packet whenever the original packet was damaged. However, error detection and packet reassemble were only done by the destination node in M-FEC. M-FEC is more efficient than MPC because retransmission can be eliminated in the intermediate nodes.

Segmented Data Reliable Transfer (SDRT) based on a hybrid Automatic Repeat Request (ARQ) and FEC approach was proposed in [14]. ARQ requires the transmitter to resend the packet in which the error has been detected. FEC uses special codes that allow the receiver to detect and correct a limited number of errors. This hybrid mechanism supports quick encoding and decoding algorithm called Simple Variant of Tornado (SVT) codes to improve the reliability more than the pure ARQ.

These approaches focused on the reliability of data transmission in the physical or the data link layer. They tried to maintain stable communications which is a base condition to collect sensing data. However, it is hard to verify the performance of these MAC-based schemes in practical applications and this causes compatibility problems. If sensor platforms use different MAC-based schemes, they cannot communicate with each other. Our approach is easy to implement, because the proposed scheme is applied to the application layer.

### 3.2. Routing Protocols

The routing protocols determine the path from a source node to a destination node. In [15], a robust location based routing scheme was proposed. To achieve the robustness and energy efficiency, a novel routing protocol called Hop by Hop Vector Based Forwarding (HH-VBF) was suggested. HH-VBF overcomes the limitations of the existing VBF such as a small data delivery ratio and high throughput fluctuations through a self-adaption procedure. That means the decision of forwarding a packet to the next node is based on hop-by-hop.

Energy Aware Routing for Real-Time and Reliable Communication (EARQ) [16] provides real-time and reliable delivery of packets by considering the energy awareness of the next path. It is one of the proactive routing protocols that maintain a routing table on each node. To achieve real-time delivery, only paths that may deliver a packet in time are selected. EARQ may send a redundant packet through an alternate path to improve the reliability.

These routing protocols aimed to improve reliability of the packet transmission at the network layer by selecting the next proper node. These studies tried to modify routing algorithms of TWSNs considering to features of UWSNs. We designed our checkpointing scheme focused on clustering based routing algorithms, because the most typical UWSN architecture is based on a hierarchical structure.

## 4. Head Node Checkpointing

### 4.1. Requirements

The head node plays an important role in coordinating its cluster and collecting data from its cluster members in clustering protocols [17]. When a failure occurs at the head node, member nodes should quickly and correctly detect the failure of the head node. If the member nodes are unaware of the failure at the head node, they send meaningless data and therefore waste energy. Some member nodes determine that a failure of the head node occurs despite the fact that the head node is operating properly. This wrong decision leads to unnecessary energy consumption due to recovery and re-clustering of the network. Therefore, it is important to reduce recovery latency and prevent the head node reelection process so as to prolong the lifetime of the network.

### 4.2. System Design

We propose a checkpointing scheme for the head node in clustering routing protocols to minimize the recovery cost and latency. The head node election phase aims to select a stable node as a cluster head and give an equal opportunity to every node to evenly distribute the node’s energy consumption. This phase involves heavy message exchanges among member nodes such as [18]. Each member node broadcasts its weight to others [(*N* − *1*)*^2^*] and the head node, which has the highest weight, sends their ID to member nodes (*N* − *1*). During the head node election step our scheme elects additional backup nodes by using the fact that head nodes notify their IDs in the end of head node election step, to checkpoint the information of a head node. The state of backup nodes is similar to the head node in terms of the residual energy or their ability to operation. All collected information sent by normal nodes to the head node is also saved in backup nodes. The backup nodes periodically detect the state of the head node. If the head node has a transient problem, one of the backup nodes that is the closest node to the head node replaces the failed head node and serves as a new head node.

**Figure 6.**
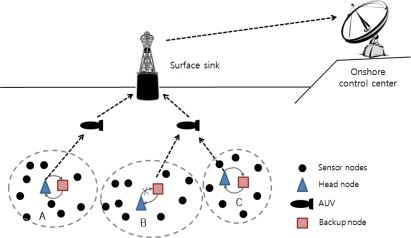
Overview of the proposed scheme.

Figure 6 shows an overview of our scheme as the checkpointing mechanism is applied to the head node. When the head node operates properly (clusters A and C), backup nodes save only the checkpoint information and monitor the state of the head node. In the case of cluster B, the head node cannot carry out its tasks when it encounters either a software or hardware problem. A backup node then operates as a head node based on the checkpointing information. Through this checkpointing scheme, we can prevent information loss caused by failure in the head node, and we can reduce the recovery latency caused by the frequent reelection of a head node.

### 4.3. Energy Consumption Model

We use a Markov model to find the minimum number of backup nodes that meets the expected reliability of users and the energy analysis model to determine the optimal checkpointing interval. The model that is designed to represent the energy consumed during the transmission of data is divided into a shallow ocean (depth of water less than 100 m) and a deep ocean (depth of water greater than 100 m) model in UWSNs. In this paper, the energy consumption of the data transmission model is based on the shallow ocean model [19]. Table 1 shows the notations and functions used in this paper to model our system.

#### 4.3.1. Assumptions

To simplify our model, we make the following assumptions:

- The reference network model based on [20]
- All nodes know their residual energy
- There is no communication error between two nodes
- All nodes are fixed (*i.e.*, we do not consider node mobility)
- The failure rate (λ) is based on a Poisson distribution

#### 4.3.2. The Minimum Number of Backup Nodes

There is a trade-off between reliability and energy consumption. As the number of backup nodes increases, reliability increases, but the energy consumption of checkpointing also increases. As a result, the lifetime of network decreases. Therefore, we find the minimum number of backup nodes that satisfies the expected level of reliability. We apply the Markov model to determine the minimum number of backup nodes when the expected reliability is specified by a user or an application designer.

**Figure 7.**
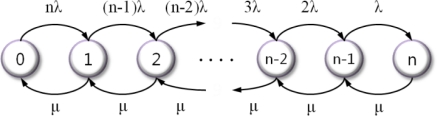
The state diagram.

In [21], there is a special case of the birth-death process that involves the use of continuous-time Markov model. Figure 7 shows the state diagram of our model, where the state refers the number of failure nodes.

If the failure rate of each node (including the head node) is λ and the repair rate is μ, the expressions for steady-state probabilities are obtained via Equations (3) and (4):

(3) $$\pi_{k}=\pi_{0}\prod_{i=0}^{k-1}\frac{\lambda (n-i)}{\mu },0\leq k\leq n$$

(4) $$\pi_{0}=\frac{1}{\sum_{k=0}^{n}\rho^{k}\frac{n!}{(n-k)!}}$$

When each node has its own repair facility, such as a watchdog timer with repair rate μ, the availability of an individual component is obtained via Equation (5) and the steady-state availability is computed via Equation (6):

(5) $$A_{\text{indiv}}=\frac{1}{1+\frac{\lambda }{\mu }}=\frac{1}{1+\rho }$$

(6) $$A_{\text{steady}}=1-\pi_{n}=1-\frac{\rho^{n}n!}{\sum_{k=0}^{n}\rho^{k}\frac{n!}{(n-k)!}}$$

When A_steady_ is equal to the expected reliability, μ is equal to the frequency of the watchdog timer. If the failure rate of each node, (λ), is given, we can define the minimum number of backup nodes (*n*) through Equation (6).

#### 4.3.3. The Optimal Checkpointing Interval

**Figure 8.**
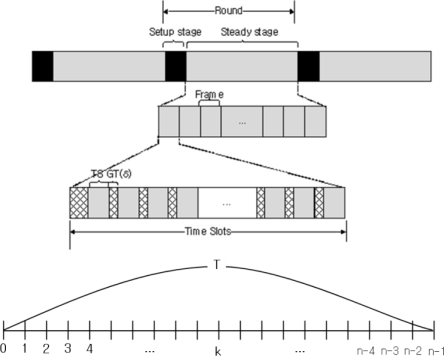
TDMA-based data collection.

Figure 8 shows the time line of the clustering protocols that use TDMA-based scheduling. Each round is composed of a setup stage and a steady stage. All clusters are formed during the setup stage, and data transmission occurs during the steady stage. A steady stage comprises several frames, and each frame is divided into time slots that are assigned to each member node so as to send data to a head node without communication interference. There is some guard time (*GT: δ*) in order to avoid acoustic collisions at the head node when two member nodes using adjacent time slots send their data message [22]. In this scheme, the head node can calculate the delay and distance to each member node through the time-of-arrival approach that measures the round-trip time of an acoustic signal between the head node and a member node [19].

If the failure rate of each node is λ, we define e^−λ T^, as there is no failure of each node during the total data collection time from all member nodes (*T*). Under this condition, the probability of failure is P_k_ = (e^−λ T^)^k–1^ (1–e^−λT^) when the head node gathers data from the k_th_ node. In underwater environments the energy consumption of data transmission between two nodes is defined by Equation (7):

(7) $$E_{\text{tx}}=S\times T_{\text{tx}}\times K,\;S=2\pi \times H\times I$$

To compare the energy consumption of our checkpointing scheme with that of an existing scheme, we define E_pre_ and E_ckpt_ as in Equations (8) and (9), respectively:

(8) $$E_{\text{pre}}=\sum_{k=0}^{N-1}\{(1-P_{k})\times E_{\text{tx}}+P_{k}\times E_{\text{elec}}\},\;E_{\text{elec}}=\{\;{\left(N-1\right)}^{2}+2(N-1)\}\times E_{\text{tx}}\;$$

(9) $$E_{\text{ckpt}}=\sum_{k=0}^{N-1}\{(1-P_{k})\times E_{\text{tx}}+P_{k}\times (N-1)\times E_{\text{tx}}\}+n\times \left\lceil \frac{k}{I_{\text{ckpt}}}\right\rceil \times E_{\text{tx}}$$

In the previous clustering routing protocol, which does not support the checkpointing mechanism, when a head node fails, this causes the election of a new head node which broadcasts the remaining energy notification messages, finds the member nodes and constructs a routing table [4].

If a head node fails at the *k*_th_ step, the difference between the energy consumption of the previous scheme and that of the proposed scheme is defined by Equation (10), as we assume that a head node failure occurs only once per round (*T*):

(10) $$P_{k}\times E_{\text{elec}}\geq P_{k}\times (N-1)+n\times \left\lceil \frac{k}{I_{\text{ckpt}}}\right\rceil ,\;I_{\text{ckpt}}>0$$

The optimal checkpointing interval is derived through Equation (11). The minimum value of I_ckpt_ that satisfies this condition is optimal.

(11) $$I_{\text{ckpt}}\geq \frac{\text{nk}}{{\left(N-1\right)}^{2}+(N-1)}$$

#### 4.3.4. The Recovery Latency

As the recovery latency is in direct proportion to the number of required messages, we compare the recovery latency of the checkpointing scheme with that of previous schemes, as shown in Equation (12):

(12) $$\begin{array}{c} D_{\text{pre}}=\sum_{k=0}^{n-1}\frac{d_{k}}{1500}+2\times \left(\frac{d_{\text{max}}}{1500}\right)+(n+1)\times \delta \\ D_{\text{ckpt}}=I_{\text{ckpt}}+\frac{d_{\text{max}}}{1500}+\delta \end{array}$$

The acoustic signal is propagated at 1,500 m/s underwater [23]. Each sensor node in a cluster sends collected data to the head node via an assigned time slot based on TDMA. The guard time (*δ*) is used to avoid collisions with the acoustic signal, and it is inserted at two consecutive time slots. The recovery latency is related to the number of messages and one of the member nodes. If the number of messages that include the failure notification packet and the control packets to reelect a new head node increase the recovery latency also increases. Increasing the number of member nodes affects the latency in the same way. The data transmission and reelection process are completed when the farthest node from the head nodes receives the acknowledgement that is used to confirm the success of data transmission between the two nodes.

## 5. Performance Evaluation

### 5.1. Experimental Environment

We evaluate our scheme in terms of energy efficiency and recovery latency. Table 2 describes the parameters used for the evaluation. The value of the parameters is based on [24], a study that investigated the transmission power and the communication latency in UWSNs.

### 5.2. Checkpointing Interval

**Figure 9.**
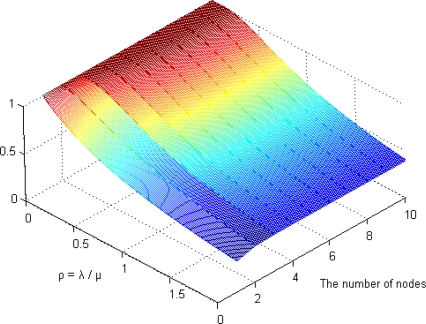
The steady-state availability.

Figure 9 shows the steady-state availability of our scheme via the number of backup nodes where ρ (λ/μ) is Equation (6). If the rate of the watchdog timer is higher than the failure rate (ρ < 1), the reliability of our system is greater than 80% when using three backup nodes. If the repair rate is identical to the failure rate (ρ = 1), our system maintains a reasonable level of availability (more than 73%) using just three backup nodes. Therefore, we determined the minimum number of backup nodes as 3.

**Figure 10.**
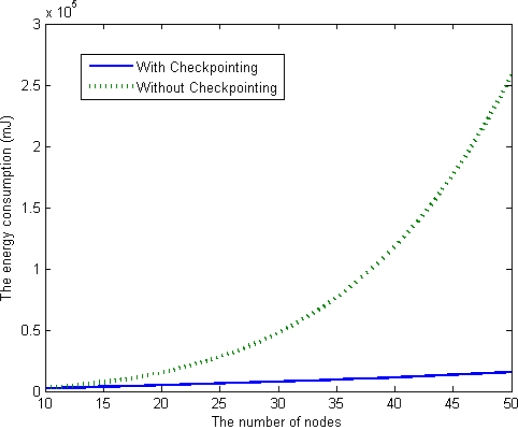
Comparison of the energy consumption values.

We simulated two schemes using MATLAB [25],a well-known simulator for verifying numerical models. We implemented our checkpointing scheme based on a clustering-based routing protocol. As shown in Figure 10, when the number of member nodes in a cluster increases in a range from 10 to 50, the average energy consumption of our checkpointing scheme is less than that of the previous scheme (without checkpointing). Our scheme is also superior to the previous scheme in terms of scalability, as the energy consumption of the proposed scheme steadily increases with the number of nodes in a cluster.

**Figure 11.**
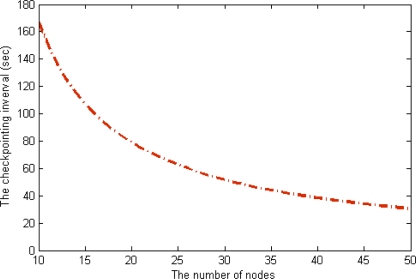
The checkpointing interval.

The energy consumption of the scheme without checkpointing is higher than that of our scheme, and the difference between the two schemes steadily increases as the number of nodes in a cluster increases. With this extra energy, our scheme reduces the checkpointing interval and increases the reliability of the underwater sensor network. In this case, we derived optimal checkpointing intervals of between about 160 s and about 30 s when the number of sensor nodes ranged from 10 to 50 in Figure 11. The results show that as the number of sensor nodes increase, the amount of extra energy (*E_pre_* – *E_ckpt_*) increases, as does the amount of checkpointing messages. In summary, the optimal checkpointing interval approaches 20 s as the number of sensor nodes in a cluster increases.

**Figure 12.**
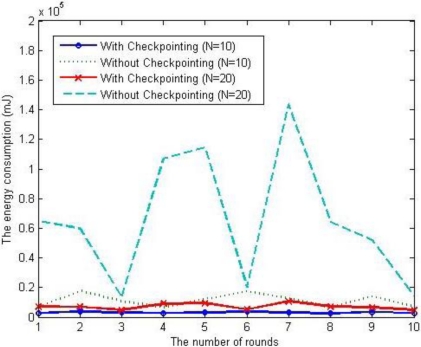
Comparison of the energy consumption values (randomized).

To verify the proposed energy consumption model, we simulated our scheme under the uniform distribution of a node failure rate. When the number of nodes is 10 and 20 respectively, the energy consumption of each scheme is shown as Figure 12. In case of previous scheme, without checkpointing, as the number of nodes increases, the energy consumption increase exponentially and the variation in each round is also fluctuating, but in the case of the proposed scheme, with checkpointing, the affection of the number of nodes is limited and the variation in each round is also small.

### 5.3. Recovery Latency

**Figure 13.**
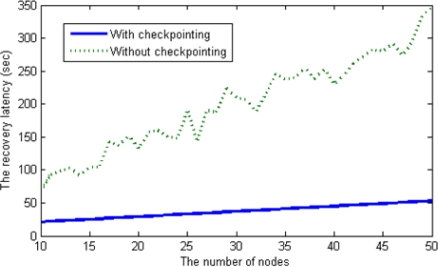
The recovery latency comparison.

To compare the recovery latency between the scheme with checkpointing and that without check-pointing, we deployed sensor nodes randomly, meaning that we assigned the distance between a head node and each member node (ranging from 1 to 15 km) arbitrarily. The recovery latency is affected by the number of messages sent during the recovery process. In the scheme without checkpointing, *O*(*n^2^*) messages are generated during the re-election process as the number of nodes increases in a cluster. However, our checkpointing scheme when applied generates only *O*(*n*)messages via the backup node; thus, the recovery latency with checkpointing increases linearly, as shown in Figure 13.

**Figure 14.**
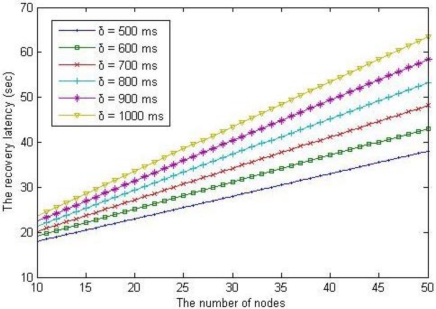
The recovery latency as the guard time (*δ*).

Figure 14 shows the variation of recovery latency as the guard time increases range from 500 ms to 1,000 ms. The guard time that prevents the collision of acoustic signals affects to the recovery latency directly. The recovery latency with checkpointing scheme is more efficient than that of without checkpointing scheme even if the guard time is up to 50 s.

## 6. Conclusions

In the design of energy efficient underwater sensor networks, it is necessary to consider the resource constraints of the sensor nodes and the underwater environment. Underwater sensor networks operate in harsher conditions than TWSNs. The applications of UWSNs are also expanding into areas that represent harsher and generally more dangerous environments. Therefore, the reliability of the network is considered to be of upmost importance.

In this paper, we proposed a checkpointing scheme for clustering routing protocols. Through our simulation results, we showed that the proposed checkpointing scheme results in an UWSN that is more energy efficient and has lower recovery latency when the head node fails than the previous schemes.

## Figures and Tables

**Table 1.** List of notations used in this paper.

**Notation**	**Description**
*N*	The number of sensor nodes
*n*	The number of backup nodes
*λ*	Failure rate of each node
*μ*	Repair rate of backup nodes
*ρ*	*λ/μ*
*E_tx_*	The amount of energy to data transmission between two nodes
*S*	The transmission power (dBm)
*d*	Distance between two nodes
*H*	The depth of water
*I*	The intensity of acoustic signal
*T_tx_*	Transmission time
*K*	Packet size
*P_k_*	The failure probability of head node at the k_th_ data transmission
*E_pre_*	The energy consumption of previous scheme (without checkpointing)
*E_ckpt_*	Energy consumption of the proposed scheme (with checkpointing)
*I_ckpt_*	Checkpointing interval
*D_pre_*	Recovery latency of previous scheme (without checkpointing)
*D_ckpt_*	Recovery latency of the proposed scheme (with checkpointing)
*δ*	The guard time
*d_max_*	Distance from the farthest sensor node to the head node

**Table 2.** Parameters for the simulation.

**Notation**	**Description**
*N*	10 ≤ *N* ≤ 50
*N*	3
*H*	50 *m*
*I*	0.061 *dB*
*T_tx_*	280 *ms*
*K*	64 *bytes*
*Λ*	1.35 × 10^−6^(0 < *λ* < 1.0)
*M*	2.7 × 10^−6^(0 < *λ* < 1.0)
*P*	0.5 (*λ/μ*)
*I_ckpt_*	25 s
*δ*	500 ms ≤ *δ* ≤ 1,000 ms
*d_max_*	15 km (the maximum transmission range of acoustic modem = about 20 km)

## References

[1] Akyildiz I., Pompili D., Melodia T. (2005). Underwater acoustic sensor networks: Research challenges. Ad Hoc Netw.

[2] Akyildiz I., Pompili D., Melodia T. (2004). Challenges for efficient communication in underwater acoustic sensor networks. ACM Sigbed Rev.

[3] Sozer E.M., Stojaovic M., Proakis J.G. (2000). Underwater acoustic networks. IEEE J. Ocean. Eng.

[4] Wang P., Zheng J., Li C. (2009). Cooperative fault-detection mechanism with high accuracy and bounded delay for underwater sensor networks. Wirel.Commun.Mob.Comput..

[5] Manjula B., Manvi S. (2011). Issues in underwater acoustic sensor networks. Int. J. Comput. Electr. Eng..

[6] Yunus F., Ariffin S., Zahedi Y. A Survey of Existing Medium Access Control (MAC) for Underwater Wireless Sensor Network (UWSN).

[7] Cayirci E., Tezcan H., Dogan Y., Coskun V. (2006). Wireless sensor networks for underwater surveillance systems. Ad Hoc Netw..

[8] Chen K., Zhou Y., He J. (2009). A localization scheme for underwater wireless sensor networks. Int. J. Adv. Sci. Technol..

[9] Cui J., Kong J., Gerla M., Zhou S. (2006). Challenges: Building scalable mobile underwater wireless sensor networks for aquatic applications. IEEE Netw.

[10] Vasilescu I., Detweiler C., Rus D. AquaNodes: An Underwater Sensor Network.

[11] Heidemann J., Ye W., Wills J., Syed A., Li Y. Research Challenges and Applications for Underwater Sensor Networking.

[12] Xiong J., Lyu M., Ng K. (2011). A reliable and efficient MAC protocol for underwater acoustic sensor networks. Int. J. Distrib. Sens. Netw.

[13] Xu J., Li K., Min G. (2011). Reliable and energy-efficient multi-path communications in underwater sensor networks. IEEE Trans. Parallel Distrib. Syst.

[14] Xie P., Zhou Z., Peng Z., Cui J., Shi Z. (2010). SDRT: A reliable data transport protocol for underwater sensor ne. Ad Hoc Netw.

[15] Nicolaou N., See A., Xie P., Cui J., Maggiorini D. Improving the Robustness of Location-Based Routing for Underwater Sensor Networks.

[16] Srimathi C., Vaideeswaran J., Kumar S. (2011). EARQ: Energy aware routing for real-time sensor networks. Int. J. Eng. Sci. Technol.

[17] Wu Z., Tian C., Jiang H., Wenyu L. Minimum-Latency Aggregation Scheduling in Underwater Wireless Sensor Networks.

[18] Chinara S., Rath S.K. Energy Efficient Mobility Adaptive Distributed Clustering Algorithm for Mobile Ad Hoc Network.

[19] Proakis J.G., Rice J.A., Stojanovic M. (2001). Shallow water acoustic networks. IEEE Commun. Mag.

[20] Domingo M.C., Prior R. (2008). Energy analysis of routing protocols for underwater wireless sensor networks. Comput. Commun.

[21] Trivedi K.S. (2002). Continuous Parameter Markov Chains, Probability and Statistics with Reliability, Queuing, and Computer Science Applications.

[22] Liu L., Xiao Y., Zhang J. A Linear Time Synchronization Algorithm for Underwater Wireless Sensor Networks.

[23] Partan J., Kurose J., Levine N. (2006). A survey of practical issues in underwater networks. Mob. Comput. Commun. Rev..

[24] Domingo M.C. (2011). A distributed energy-aware routing protocol for underwater wireless sensor networks. Wirel. Pers. Commun.

[25] MathWorks http://www.mathworks.com/.

